# Recent insights into HSP70: proteostasis and beyond

**DOI:** 10.3389/fmolb.2026.1791536

**Published:** 2026-04-23

**Authors:** Kristina Pustovaya, Artem Venediktov, Vladislav Soldatov, Egor Kuzmin, Ksenia Pokidova, Viktoria Gartzeva, Olga Payushina, Vassiliy Tsytsarev, Igor Meglinski, Gennadii Piavchenko

**Affiliations:** 1 Human Anatomy and Histology Department, I. M. Sechenov First Moscow State Medical University (Sechenov University), Moscow, Russia; 2 Magnetic Resonance Imaging Laboratory, CIC biomaGUNE, Parque Cientifico y Tecnologico de Gipuzkoa Paseo Miramon, Donostia/San Sebastian, Gipuzkoa, Spain; 3 Whiting School of Engineering, Johns Hopkins University, Baltimore, MD, United States; 4 College of Engineering and Physical Sciences, Aston University, Birmingham, United Kingdom

**Keywords:** GRP78, HSC70, HSPA1A, molecular chaperones, mortalin, protein quality control

## Abstract

Since the 1980s, 70 kDa heat shock proteins (HSP70s) have been recognized as central regulators of proteostasis, with diverse roles in cellular physiology and pathology. Recent research has significantly expanded our understanding of these molecular chaperones, revealing functions that extend beyond their classical roles in proteostasis. In this review, we integrate these emerging insights with foundational knowledge by outlining the biology of HSP70s, with particular emphasis on recent discoveries, such as new data on the substrate specificity and molecular dynamics of HSP70–client interactions. In addition, increasing evidence highlights their noncanonical anti-inflammatory properties, as well as other nonimmune functions, including the promotion of adipose tissue browning and the enhancement of angiogenesis through extracellular HSP70 activity. Finally, although HSP70s have long been known to regulate mRNA degradation in a transcript-specific manner, new findings demonstrate their ability to bind double-stranded RNA, further broadening their functional repertoire.

## Introduction

Molecular chaperones are cellular components that maintain the integrity of proteome and facilitate proper folding, maturation, and recycling of proteins (Zuppini et al., 2025). Many molecular chaperones are heat shock proteins (HSPs), as they are essential in stress conditions, including temperature-related (Hagymasi et al., 2022). Within this group, the 70-kDa HSP family (HSP70 or HSPA) is crucial in safeguarding protein homeostasis or proteostasis (Mecha et al., 2022; Binder and Pedley, 2023).

The HSP70 family comprises the largest number of members whose functions differ, although with shared patterns of proteostatic activity (Kampinga et al., 2009; Soldatov et al., 2024). Although distinct HSP70s are localized in the mitochondria and endoplasmic reticulum (ER), the principal HSP70 family members are located in the cytosol. These include HSPA8, also known as heat shock cognate protein (HSC70), which is constitutively expressed in the cytosol, and HSPA1, which has two stress-inducible isoforms, HSPA1A and HSPA1B (Kampinga et al., 2009; Venediktov et al., 2023). The structures of HSC70 and HSPA1 are highly similar, and they share most co-chaperones; however, their functional machinery and client protein repertoires differ (Ryu et al., 2020). By managing these client proteins, HSP70s function in two principal modes: facilitating the proper folding of nascent and partially denatured proteins or targeting abnormal and irreversibly damaged proteins for degradation and clearance, a process known as protein quality control (PQC) (Yan et al., 2020).

HSP70s deploy chaperone functions either through an ATP-dependent mechanism, known as foldase activity, which facilitates protein folding (Mayer and Bukau, 2005), or via an ATP-independent mechanism that prevents protein misfolding, referred to as holdase activity (Karunanayake and Page, 2021). In addition to their molecular chaperone roles, HSP70s are involved in various other cellular processes, including the regulation of programmed cell death (Venediktov et al., 2023). Owing to their high intracellular abundance, they also serve as indicators of compromised cellular integrity: once released extracellularly, HSP70s act as damage-associated molecular patterns (DAMPs), triggering immune responses through receptors such as the toll-like receptors TLR2 and TLR4 (Theivanthiran et al., 2022; Wang H. et al., 2025; Hulina et al., 2018). This ubiquitous role reflects their profound integration into multiple signaling and metabolic pathways, making HSP70s a frequent hot spot in pathological conditions, including neurodegeneration, cancer, and inflammation (Craig and Marszalek, 2017; Martinková et al., 2018). Recent evidence shows that HSP70s are involved into transmembrane protein translocation via entropic pulling (Rukes et al., 2024), into the protection of damaged muscle fibers via calcium reuptake from the sarcoplasm to the reticulum (Barfoot et al., 2025), and into the regulation of enzymes that mark proteins with ultraviolet-dependent damage (Zeng et al., 2025). New data have emerged on the role of HSP70s in the regulation of inflammation (Borges et al., 2025), amyloid accumulation (Ruggiero et al., 2025), vascular endothelial function (Pinto-Martinez et al., 2026) and adipose tissue (Zhuang et al., 2025), as well as its effect on viral replication by binding double-stranded RNA (Fletcher et al., 2025). Overall, we aim to synthesize the expanding body of recent research with classical studies (Kao et al., 1985; Subjeck et al., 1985) on HSP70s, providing an integrated perspective that highlights both foundational discoveries and emerging insights.

## General overview of HSP70s

### Members

The term HSP70 refers to a family of chaperones with a molecular weight of approximately 70 kDa, consisting of 13 members in humans with distinct functions and intracellular localizations (Kampinga et al., 2009), derived from corresponding genes, whereas HSPA7 is usually considered as a pseudogene (Ding et al., 2021), with ambiguous data on HSPA7 role (Li et al., 2022). HSPA4, despite its traditional name, actually belongs to the HSP110 family (Kaneko et al., 1997). Together with the HSP110 family, the HSP70 family is sometimes referred to as the HSP70 superfamily (Kampinga et al., 2009). A general overview of each member is provided in Table 1 and Figure 3, whereas new insights about certain members are discussed in Section *Distinct HSP70s*.

**TABLE 1:** Members of the HSP70 family. HSP70s may be stress inducible or not inducible and reside in different cell sites at different concentrations, thereby affecting their function.

Family member	Localization	Key features & functions	References
HSPA1A & HSPA1B	Cytoplasm, nucleus, plasma membrane, exosomes	Inducible chaperone with two similar isoforms to prevent misfolding under stress conditions, multifaceted interactions with other chaperones in proteostasis, early recompartmentalization to nucleolus in response to heat stress, immune signaling in cell damage	Calderwood et al. (2016), Deane and Brown (2017), Shevtsov et al. (2018), Xiao et al. (2025), Zuo et al. (2025)
HSPA1L	Cytoplasm, nucleus	Non-inducible and low abundant HSPA1 isoform, promotes translocation of certain damaged proteins from organelles	Hasson et al. (2013)
HSPA2	Cytoplasm, nucleus, exosomes, extracellular vesicles	Cellular differentiation as well as signaling during differentiation and response to cell damage	Sojka et al. (2023), Gogler et al. (2025)
HSPA3	Excluded		
HSPA4, HSPA4L	Belongs to the HSP110 family		
HSPA5/GRP78/BiP	Endoplasmic reticulum, exosomes	Endoplasmic reticulum–stress and cell cycle control	Hetz et al. (2020); Du et al. (2025)
HSPA6	Cytoplasm, perinuclear zone, exosomes	Inducible chaperone with late relocalization to nucleolus in response to heat stress	Deane and Brown (2017)
HSPA7	Pseudogene		
HSPA8/HSC70	Cytoplasm, nucleus, cell membranes	Key actor in protein quality control, especially in chaperone-mediated autophagy and chaperone-assisted selective autophagy	Calderwood et al. (2016), Qiao et al. (2023), Ulbricht et al. (2013)
HSPA9/mtHSP70/GRP75/mortalin	Mitochondria	Maintenance of mitochondrial proteostasis and electron transporting chain components assembly, especially proteostasis in damage by reactive oxygen species	Song et al. (2023), Bakovic et al. (2025), Acquarone et al. (2025)
HSPA10	Belongs to the HSP110 family		
HSPA11	Non-existent		
HSPA12A	Cytoplasm	Proteostasis in proteins participating in metabolic regulation	Han et al. (2003), Yu et al. (2024)
HSPA12B	Cytoplasm, exosomes	Endothelial isoform, immune signaling in endothelial damage	Radons (2016), Fan et al. (2020)
HSPA13	Endoplasmic reticulum, exosomes	Modulation of protein translocation, control of nascent proteins	Espinoza et al. (2022)
HSPA14	Cytoplasm, plasma membrane	Inducible chaperone with assistance in proper folding during protein translation	Venediktov et al. (2023), Radons (2016)

### Structure and mode of action

**FIGURE 1:**
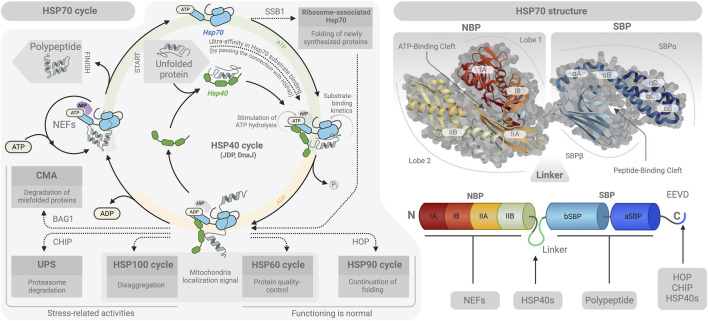
Molecular structure of HSP70 and its cycle. The structure of HSP70 (on the right) includes a nucleotide-binding domain (NBD) for binding to NEF, a substrate-binding domain (SBD) for binding to polypeptides, a linker for binding to HSP40, and a C-terminal domain for interaction with co-chaperones HOP, CHIP or HSP40. Classical protein folding (on the left) involves the interaction of HSP40 and HSP70 with the participation of ATP. However, protein formation in ribosomes without the participation of HSP40 is also possible. Folding proteins with a more complex structure require the participation of NEFs, co-chaperones, HSP90, or HSP110. If protein folding is impossible, the aberrant molecule is destroyed by UPS or HSP100-associated disaggregation. Created with BioRender.

A molecule of HSP70 consists of an N-terminal nucleotide-binding domain (NBD), a substrate-binding domain (SBD), a linker region connecting the two, and variable C-terminal motifs such as EEVD (Flaherty et al., 1990) (Figure 1). In turn, the SBD includes two functional parts: a β subdomain of 8 β chains and an α subdomain of 4–5 α chains (Zhu et al., 1996; Stevens et al., 2003). The substrate binding by the SBD is implemented in two steps: after binding to the client protein via SBD-ß, HSP70s trap it by closing SBD-α, serving as a cap or lid (Wang et al., 1998; Zhang et al., 2014).

Generally, HSP70 binding sites in polypeptide chains repeat every ∼36 residues, mainly in β-sheets with four to five residues, such as leucine, isoleucine, valine, phenylalanine, and tyrosine (Rüdiger et al., 1997). The following ATP‒ADP transition in the NBD “closes” the flexible double-hinged lid of the SBD, preventing the client protein from leaving (Marszalek, 2022; Kumar et al., 2023). After the binding of a new ATP molecule in the NBD, the client protein leaves the substrate binding pocket (Qi et al., 2013). The cycles of binding and release can be repeated multiple times, after which the substrate is either released into the cytoplasm to exert its functions or transferred to other chaperone machines, such as Hsp90 (Lang et al., 2021).

HSP70s exert three major activities on client proteins: 1) preventing nascent proteins from misfolding and facilitating their proper folding; 2) preventing aggregation of mature proteins; and 3) solubilizing or refolding aggregated proteins (Mayer and Bukau, 2005). When interacting with nascent and mature proteins, HSP70s bind hydrophobic patches via the SBD, thus preventing spontaneous lipophilic cross-interactions. When acting on aggregated proteins, HSP70s perform the same mechanism of binding to exposed hydrophobic patches, allowing for reassembly of the compromised structure (although details have yet to be understood mechanistically). For this mechanism, HSP70s recruit different co-chaperones for specific activities: e.g., in mammals, HSP40 is involved in folding, and HSP40/HSP110 are involved in refolding (Mauthe et al., 2025a; Mauthe et al., 2025b). These interactions are probably species-specific. Thus, human HSP70s, apparently, fail to recognize client proteins of other species, for example, of *Escherichia coli* (Ambrose et al., 2024).

New details of molecular HSP70 action have recently been observed by an *in silico* research (Mahto et al., 2024). Previously, it had been reported about the structures that allow lid opening in SBD when releasing the substrate (PDB 4JN4) (Qi et al., 2013). Mahto and colleagues have revealed the lid opening to be greater than anticipated. In addition, recent data elucidate the physical nature of the binding of client proteins to HSP70 during their translocation across biological membranes. Essentially, proteins synthesized in the cytoplasm must unfold to pass through compact membrane channels to enter organelles, and the process of subsequent refolding requires the assistance of HSP70s. The conventional explanation of how HSP70s bind to unfolded client proteins that escape channels involves three disputing theories: 1) the Brownian ratchet theory, which suggests that a passive HSP70 plays a role in enveloping client proteins and limiting their movement; 2) the power stroke theory, which proposes that a strong transformation of an HSP70 molecule to a client protein occurs; and 3) the entropic pulling theory, which postulates that HSP70 increases entropy via client protein binding and therefore moves forward to obtain a more thermodynamically appropriate conformation. A recent study (Rukes et al., 2024) provided unambiguous evidence supporting the Entropic Pulling theory. Using an elegant approach based on biological nanopore sensors incorporated into artificial lipid membranes, the authors monitored the escape of various substrates from the pore. Despite opposing electric forces that hinder substrate escape, the presence of HSP70 significantly facilitates translocation by pulling the substrate to the opposite side of the membrane (Rukes et al., 2024).

In addition, HSP70s begin protecting proteins from misfolding as soon as the first segments of the polypeptide chain emerge from the ribosome. However, possible mistranslation, which is caused by mutations in tRNA genes, may alter the protective activity of HSP70 on nascent proteins (Lant et al., 2018). Recently, McDonald and colleagues revealed that frequent mistranslation events involving a shift from serine to either proline or arginine have distinct effects: serine–to-proline substitution reduces the ability of polypeptide chains to bind HSP70s, whereas serine–to-arginine substitution prevents misfolded nascent proteins from being denatured and degraded (McDonald et al., 2025).

### Regulation of HSP70 levels

HSP70s likely bear the primary burden of cellular adaptation to various stress factors, more so than other molecular chaperones do. For example, under sustained heat stress, HSP70 levels increase more significantly than those of other heat shock proteins (Albokhadaim, 2025). However, HSP70 expression reflects adaptation not only to external environmental conditions but also to intrinsic factors, such as age. For example, human studies have shown a marked decline in HSP70 levels in older individuals (Tandara et al., 2006; Rea et al., 2001), what is recently approved in ruminants (Kaushik et al., 2022).

**FIGURE 2:**
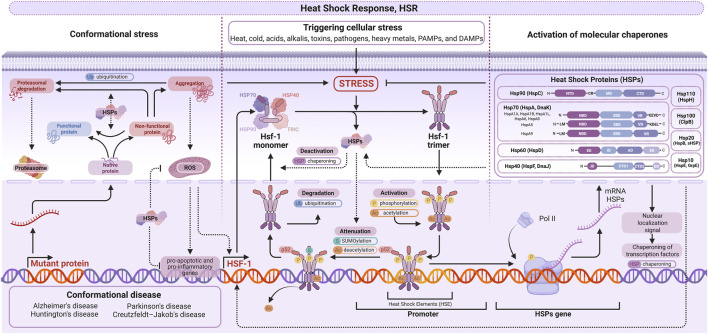
HSF1/HSP70 axis. At the center, cellular stress signalization by pathogen-, damage-, and microbial-associated molecular patterns, as well as chemical and physical factors, affects heat shock factor 1 (HSF1), which experiences trimerization and binds to heat shock elements (HSEs) on promoters for multiple chaperones. Trimerized HSF1 is destined for proteasomal degradation after ubiquitination by ligases or for monomerization. Moreover, synthesized chaperones have various biochemical structures (on the right) and functions (on the left), regulating HSF1 levels, apoptosis, and protein quality control. Created with BioRender.

However, influenced by various signaling pathways, HSP70 upregulation is driven primarily by heat shock factors, particularly HSF1, which are involved in a complex regulatory network (Figure 2). HSF1 is a transcription factor that undergoes trimerization and multiple posttranslational modifications, including phosphorylation, in response to heat stress. Upon activation, HSF1 trimers translocate to the nucleus and bind to heat shock elements (HSEs) in the promoters of target genes, thereby increasing the transcription of HSP70s, which then undergo degradation or re-monomerization (Vihervaara and Sistonen, 2014). Importantly, even though HSF1-driven HSP70 upregulation is mediated by gene expression regulation, at the whole-cell level, HSP70 tends to be distributed unevenly across the cell in a demand-dependent manner. For example, a recent study revealed that oxidative damage following ischemic assault resulted in the upregulation of HSP70, specifically in astrocyte endfeet (Shim et al., 2025).

HSF1 serves as a sensor of various modalities that detect disturbances in homeostasis and activate HSP expression. For example, exposure to physical stimuli of suprathreshold intensity—such as heat or mechanical forces (Gong et al., 2012) — as well as oxygen deprivation (Shim et al., 2025), can upregulate HSF1 expression. Multiple biochemical pathways that modulate HSF1 activity have also been identified. Sirtuin 1 (SIRT1) and the insulin-like growth factor receptor (IGFR) are among the most pharmacologically relevant, although not exhaustive, examples of stimulators of the HSF1–HSP70 axis (Vihervaara and Sistonen, 2014; Westerheide et al., 2009). Transient receptor potential vanilloid 1 (TRPV1) — primarily known for mediating high-temperature sensation—also directly regulates HSF1. This enables TRPV1-mediated regulation of HSP70 by capsaicin (Bevan et al., 2014) or, less classically, by cannabidiol (Ma et al., 2025). In addition, HSF1 may act directly in proteostasis without upregulating HSP70, as there is evidence of HSF1 binding to defective proteins such as amyloid oligomers (Tang et al., 2020).

### Intrinsic control of HSP70 activity

In addition to the levels of HSP70s, their biochemical activity may also be finely tuned to adapt to cellular demands. In addition to the presence of ATP and natural co-chaperones such as HSP40s, this activity may be strongly activated or inhibited, principally affecting the efficiency of HSP70-driven proteostasis. Recently, new efforts have been made to design molecular constructs that mimic or replace co-chaperones, thereby increasing HSP70 activity (Zhang et al., 2025).

HSP70s may control their own activity by changing conformation or joining into an oligomeric structure. The foldase activities of HSP70s require ATP, which binds in a Mg^2+^- and K^+^-dependent manner (Bercovich et al., 1997; Mas and Hiller, 2025). Nevertheless, the content of ionized calcium also affects the rate of ATP usage, at least for HSP70s in the ER (Mas and Hiller, 2025). Importantly, the kinetics of foldase activity depend on the type of nucleotide exchange factor (NEF) employed to provide the ATP‒ADP transition. These factors also affect the functions of certain HSP70s but have different degrees of affinity for HSP70s. For example, among the NEFs, BAG3 affinity for HSP70s is the highest, with a lower affinity for BAG1, followed by HSP110 and BAG2 (Rauch and Gestwicki, 2014). In addition to the electrolytic content and NEF involvement, the monomeric/oligomeric shift of HSP70 also affects the mode of ATP recruitment. Certain cellular activities, such as clathrin removal by HSC70, require a trimeric HSP70 (Coimbra et al., 2025).

## Distinct HSP70s

### HSPA1A/B and HSPA8/HSC70

**FIGURE 3:**
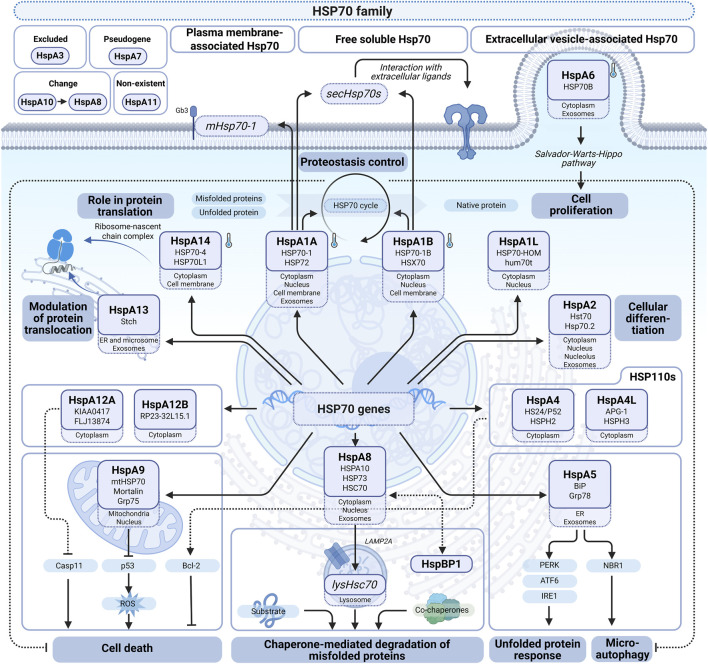
HSP70 family members. At the center, HSP72 (HSPA1A, inducible HSP70) and HSP73 (HSC70/HSPA8, constitutive HSP70) account for principal HSP70s, reflecting key mechanisms to prevent misfolding and to provide protein quality control by proteasomal degradation, optionally replaced by autophagy. Lower right/left, respectively: HSPA5 (GRP78/BiP), ER-related HSP70, and mitochondrial HSP70 (mortalin/HSPA9), both of which are stress inducible and encoded by nuclear DNA, control proteostasis associated with corresponding organelles. “Minor” HSP70s are found in relatively low amounts, although they take part in vital functions such as growth regulation via the Salvador-Warts-Hippo pathway by HSPA6. Upper left: changes in the conventional HSP70 classification. Created with BioRender.

Although the HSP70 family generally includes many members (Table 1; Figure 3), its basic actors, constitutive HSPA8 (also known as HSC70 and HSP73) and isoforms of inducible HSPA1, carry out most of the functions of the cytosolic response to protein misfolding or aggregation. Although HSPA1A (also known as HSP72) and HSPA1B differ in several respects, they can respond in a coordinated manner to cellular damage, as recently demonstrated in the cardiac muscle tissue of C57Bl/6 mice exposed to black carbon (Zuo et al., 2025). However, our current understanding of HSPA1 remains limited—likely just the tip of the iceberg—as emerging evidence suggests that it may participate in numerous, previously unrecognized molecular pathways. For example, recent findings indicate that HSPA1B facilitates the exosomal secretion of metalloproteinases from macrophages, thereby helping to mitigate fibrosis (Xiao et al., 2025). Notably, this study did not investigate the role of HSPA1A.

Inducible HSPA1 plays a critical role in maintaining cytosolic protein stability under stress conditions (Yenari et al., 1999). For example, in contracting skeletal muscle tissue, the sarcoplasmic reticulum Ca^2+^-ATPase (SERCA) facilitates calcium reuptake into the reticulum, preventing calcium overload in the sarcoplasm—likely in synergy with HSPA5 (Mázala et al., 2024). SERCA has already been shown to remain functionally intact following heat-induced damage owing to the protective activity of HSPA1 (Fu and Tupling, 2009). Recently, murine SERCA was also reported to mediate calcium reuptake from the sarcoplasm in mechanically damaged tissues after heat stress through an HSPA1-dependent mechanism (Barfoot et al., 2025).

In addition to stabilizing cytosolic proteins, HSPA1A also protects proteins of membrane organelles from damage. Recently, it was shown that the pathogenicity of mycobacteria in tuberculosis may involve the destruction of HSPA1A and its routing to proteasomal degradation. This occurs through noncatalytic stimulation by cytosolic cis-aconitate decarboxylase 1, which is upregulated in response to mycobacterial infection (Yang et al., 2025).

Some proteins are stabilized by both HSPA1 and HSC70. Initially, these chaperones together provide a proper folding of nascent proteins closer to 80S ribosomal subunit in eukaryotes (Han B. et al., 2025). Further, in cytosol, they also maintain the stability of protein kinase B (AKT), a key regulator of cell survival (Koren et al., 2010). However, because AKT is often overactivated in cancers, this chaperone-mediated stability can contribute to pathological processes. Recently, the circadian clock gene Period2 was shown to be overexpressed, and its protein product inhibits the binding of HSP70 to AKT (Yu et al., 2025). Thus, the PER2-dependent mechanism disrupts AKT proteostasis, potentially altering cell fate.

Despite the prominent role of HSPA1 in protecting proteins from damage, many autophagic processes require the suppression of HSPA1 activity. Earlier studies proposed that macroautophagy, regulated by p62, is accompanied by increased activity of most HSP70s (Sheng et al., 2012). However, heat stress-induced upregulation of HSF1 and HSP70 silences macroautophagy stimulators such as mitogen-activated protein kinase (MAPK) through mechanisms involving the mammalian target of rapamycin (mTOR) pathway (Alhasan et al., 2024), as demonstrated in cell culture models. Consistently, activation of macroautophagy is accompanied by decreases in HSPA1 and HSPA5 (ER-associated HSP70) levels (Sattari et al., 2025).

In contrast to HSPA1, HSC70 is more prone to provide PQC and is capable of maintaining proteome stability via both protein routing to proteasomes and autophagy. Generally, ensuring solubilization and preventing misfolding is a primary event of HSP70 activity, whereas the ubiquitin–proteasome system (UPS) is a compensatory mechanism to degrade proteins that are irreversibly damaged or become damaged at excessive levels unable to be refolded; autophagy is the next line of compensation active when the UPS cannot degrade proteins, especially during senescence (Feleciano et al., 2019). Thus, the UPS is a vital first-line component of PQC, and HSC70 is well known to recruit it when it joins its co-chaperone, CHIP (Shimura et al., 2004; Zhang et al., 2020). Importantly, HSPA1 can be degraded by the UPS, and CHIP blockade prevents this degradation, which is a useful tool for slowing the pace of cell death (e.g., in cardiovascular pathology) (Lin et al., 2025).

HSC70 has been recently shown to ensure proper interaction between S-phase kinase-associated protein 1/cullin 1/F-box protein (SCF) and constitutive photomorphogenesis 9 signalosomes (CSNs) (Nishimura et al., 2025). This interaction marks regulatory enzymes for ubiquitination if they are damaged by physical factors, especially ultraviolet light or radiation (Lyapina et al., 2001). For example, the stability of HSP90 molecules is provided by the SCF–CSN machinery (Zeng et al., 2025). This role of HSC70 clearly contributes to the overall functioning of the UPS.

Despite this “multifaceted hiring” in PQC, HSP70s, which represent the most common group of diseases associated with gradually worsening proteostasis, have long been considered promising tools for hindering neurodegenerative pathology. Unfortunately, the efficacy of elevated HSP70 levels and/or activity is much more evident *in vitro* and *in vivo* than in clinical studies, as we noted earlier (Venediktov et al., 2023). For example, in amyotrophic lateral sclerosis (ALS), HSP70s may modulate mutant proteins such as SOD1, FUS, C9orf72, and TARDBP by preventing their solidification or facilitating their disaggregation and/or clearance via autophagy. Recently, Takeda and colleagues reported that mutant HSC70 — nominally beneficial for modifying the SOD1–ALS phenotype—paradoxically exacerbated symptoms in mice despite reducing SOD1 content (Takeda et al., 2025). However, our recent research revealed another mode of HSP70 involvement. Briefly, mice exhibiting the FUS–ALS phenotype (characterized by FUS translocation from the nucleus to the cytoplasm) demonstrated longer lifespan, reduced disease severity and improved histological patterns when intracellular HSPA1A was overexpressed (Piavchenko et al., 2024; Piavchenko et al., 2025a; Piavchenko et al., 2025b). These findings suggest that, compared with HSC70, HSPA1 may have a stronger protective effect in this context, although differences in the affinities of SOD1 and FUS for certain co-chaperones—and thus distinct PQC strategies—may also play a role.

### HSPA2

HSPA2, previously considered a relatively minor member of the HSP70 family, is now recognized as playing key roles in cell growth and mitosis within epithelial tissues, as well as participating in extracellular signaling (Sojka et al., 2023). Recently, Gogler and colleagues demonstrated that HSPA2 is a crucial factor in keratinocyte differentiation and migration to the strata spinosum and granulosum (Gogler et al., 2025). Moreover, their research revealed that HSPA2 knockout (KO) induces a proinflammatory cytokine secretion profile, accompanied by increased expression of receptors involved in antigen presentation.

### HSPA5/GRP78/BiP

The accumulation of unfolded or misfolded proteins in the ER activates a signaling pathway known as the unfolded protein response (UPRER), which is regulated by three main sensors: protein kinase RNA-like ER kinase (PERK), inositol-requiring enzyme 1α (IRE1α), and activating transcription factor 6 (ATF6). These three regulators, especially PERK and IRE1α, closely interact with HSPA5, or glucose-regulated protein 78 (GRP78) (Hetz et al., 2020), which is a crucial ER-associated HSP70 family member. Thus, GRP78 is extremely important for the export of proteins from the cell. For example, GRP78 has been recently reported as a key chaperone preventing misfolding of coagulation factor VIII; therefore, its stability is pivotal in hemophilia type A molecular pathology (Srivastava et al., 2025).

However, GRP78 can be transferred to mitochondria and lysosomes, especially via ER-adjacent portions of their membranes, and can be transported to the cytosol to be secreted from cells. In addition, GRP78 is normally located in the ER at low levels, and its expression (but not its functional rate) may be upregulated by calcium ionophores, calcium depletors or chelators, and inhibitors of the protein secretory pathway (Casas, 2017). Selective inhibitors of GRP78 found *in silico* with possible benefits in ER stress-related tumor treatment (Ambrose et al., 2023). Nanobodies with targeted immunotoxin delivery have recently been reported to successfully suppress GRP78, too (Wang H. et al., 2025).

Human GRP78 activity has also been shown *in vitro* to be upregulated by its posttranslational modification with cell filamentation protein (FIC) (Sanyal et al., 2015). Consistent data were obtained by Truttmann and colleagues for orthologs of GRP78 (HSP3 and HSP4) and FIC (FIC-1) in *Caenorhabditis elegans* (Truttmann et al., 2016). However, the same team has recently reported a FIC KO to improve the PQC in the ER of *C. elegans* (Van Pelt and Truttmann, 2025). Moreover, in this work, Van Pelt and Truttmann mentioned an HSP70 member of the nematodes, F44E5.4 (initially cytosolic), to manage PQC in the ER in depletion of GRP78 orthologs, at least for the clearance of mutant polyglutamine proteins.

Thus, cytosolic HSP70 may affect proteostasis in the ER in the absence of active GRP78 isoforms, although the distinct mechanisms involved remain to be elucidated. However, the aforementioned involvement of cytosolic F44E5.4 in ER-related proteostasis in *C. elegans* may be not applicable to humans, as the ER–HSP70 systems of the two species differ greatly, at least because GRP78 is the only ER-associated HSP70 in humans, although it has two orthologs in *C. elegans*. Moreover, some points of the overall machinery are similar. Both *C. elegans* and *Homo sapiens* are able to translocate ER proteins for lysosomal eradication via macroautophagy via GRP78-IRE1α mediation of the UPRER and further recruitment of translocon Sec-62 (in worms, an orthologous C18E19.2) (Fumagalli et al., 2016; Urban et al., 2025).

In addition, GRP78 activity is related to the regulation of the cell cycle. Recently, Du and colleagues demonstrated that cyclin-dependent kinase 1 (CDK1) inactivation at the end of mitosis enhances GRP78-mediated proteostasis, especially via the UPS, in epithelial cells from breast tumors (MCF10A line). In addition to regulating the cell cycle, the rate of autophagy also influences GRP78 activity (Du et al., 2025). Specifically, recent studies in a model of ischemia/reperfusion injury in mice demonstrated that GRP78 activity was suppressed by the overexpression of p62, a macroautophagy driver (Quan et al., 2025). Moreover, p62-related stimulation of macroautophagy prevents protein routing to the UPS (Liu et al., 2016). In addition, p62 recruits kelch-like enoyl-coenzyme A hydratase-associated protein 1 (KEAP1) for proper autophagosome formation; in suppressed p62, KEAP1 is known to increase cell growth and resistance to ROS via nuclear factor erythroid 2-related factor 2 (NRF2) activation (Tkachev et al., 2011; Ichimura et al., 2013). Therefore, a higher rate of damaged protein routing to macroautophagy is accompanied by lower productivity of the cytosolic UPS but increased GRP78 function and NRF2-mediated effects at the same time.

### HSPA9/mortalin/mtHSP70/GRP75

HSPA9, also known as mortalin, is a constitutive but inducible mitochondrial chaperone involved in multiple functions related to proteostasis and apoptosis. For example, ATP synthase—a vital mitochondrial enzyme—requires HSPA9 for the proper assembly of its motor components, F_0_ and F_1_. In addition, HSPA9 helps prevent the degradation of these components (Song et al., 2023).

HSPA9 levels sharply increase in response to mitochondrial damage, such as excessive reactive oxygen species (ROS) production. Elevated HSPA9 expression has recently been confirmed in patients with heart failure, particularly in those with poorer prognoses (Bakovic et al., 2025). In contrast, age-related mitochondrial changes are associated with reduced HSPA9 expression and decreased ER–mitochondria membrane coupling, leading to impaired protein degradation and diminished mitochondrial calcium uptake (Acquarone et al., 2025).

### HSPA12B

HSPA12B is an endothelium-specific isoform of the HSP70 family (Han Z. et al., 2003). Its function has been shown to be agonistic with endothelial nitric oxide synthase (eNOS) (Li J. et al., 2013). Although normally cytosolic, HSPA12B can be released from endothelial cells upon damage, where it promotes the acquisition of a pro-regenerative phenotype in macrophages via TLR4 signaling (Doan et al., 2009) and the PI3K–AKT–mTOR pathway (Zhou et al., 2020). The regenerative nature of this response has been further clarified in a recent study: Wang and colleagues reported that HSPA12B is internalized by macrophages through endocytosis, subsequently downregulating TLR4 signaling (Wang Y. et al., 2025). Thus, HSPA12B appears to act through both eNOS activation and TLR4 modulation, likely contributing to the mitigation of tissue damage.

## HSP70 interactome

### HSP40/DNAJ

**FIGURE 4:**
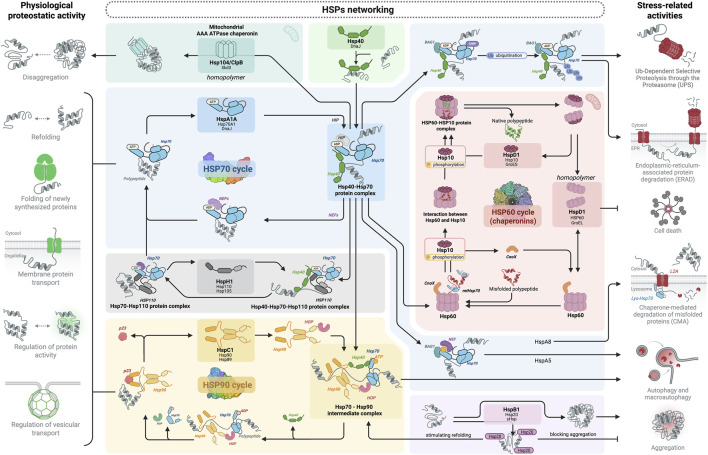
HSP70 interactome. HSP70 molecules (at the center from the left) include a nucleotide-binding domain NBD) for their foldase activity, which is ruled by co-chaperoning via HSP40s, and a substrate-binding domain (SBD) with a lid blocking client proteins from preterm leaving. HSP70s function in the refolding of previously misfolded polypeptide chains on their own, as well as in close interaction with large HSPs (above), HSP90s (below) via the HSP70/HSP90 organizing protein (HOP), and HSP60s (at the center from the right). Created with BioRender.

Molecular chaperones such as HSP40s, or DNAJs, assist HSP70s in their foldase activity (Venediktov et al., 2023) (Figure 4). Traditionally, these co-chaperones are thought to interact with HSP70 molecules solely via the N-terminal J domain of HSP40s, without the involvement of other regions—particularly the C-terminal domain and the intermediate glycine/phenylalanine-rich (GF) linker region. However, recent data revealed that the GF region also contributes to HSP70 binding, influencing the kinetics of HSP70-driven reactions—at least for HSC70 (Hobbs et al., 2025). Despite this, HSP40s are highly diverse, and certain HSP40s serve distinct client proteins while cooperating with the same HSP70 isoform, typically HSC70 (Kampinga and Craig, 2010), thereby conferring functional specificity to HSP70s (Bhattacharjee et al., 2025; Wu et al., 2025). For example, routing toward protein clearance is mediated by HSP70 in cooperation with DNAJB6, as synthesized from multiple studies in a recent review (Hentze et al., 2025). Liquid-liquid phase separation is affected by HSP70/HSP40 interaction as well as protein clearance does (cytosolic translocation of TDP-43) (Yeo et al., 2025).

Additional data on the role of HSP40s as HSP70 interactors emerged this year. Jiahui and colleagues reported that a DNAJC subfamily member, T-cell activation inhibitor in mitochondria (TCAIM), provides posttranslational modification and protection to α-ketoglutarate dehydrogenase in an HSPA9-dependent manner (Jiahui et al., 2025), revealing a novel mode of mitochondrial chaperoning. Until recently, molecular biology has regarded TCAIM primarily as an immune receptor, with secondary functions related to lipid metabolism (Korda, 2025).

### HSP90, HOP, and GRP-E

A separate family of molecular chaperones with multiple functions, HSP90, comprises well-known counterparts of HSP70s, often concurrent with them for protein binding (especially in the UPS) and even controversial in various pathology (Soldatov et al., 2024; Evans et al., 2010). However, HSP70s and HSP90s interact mainly via HSP70/HSP90 organizing protein (HOP) or CHIP (Evans et al., 2010; Donnelly et al., 2013; Ciechanover and Kwon, 2017), with HOP preventing the UPS or ER-associated protein degradation and CHIP promoting it. Interestingly, the bifurcation between HOP and CHIP apparently depends on the phosphorylation of the C-terminus of HSP70, with the phosphorylated state preferred for HOP binding (Stewart et al., 2025); an additional HSP70/HSP90 interactor, HIP, can join the N-terminus (NBD) and is not concurrent with either HOP or CHIP. A growing body of evidence supports a consideration that tetratricopeptide repeat proteins with carboxylate clamps are also required for a normal HSP70–HSP90 interaction, bringing both of them geometrically closer to client proteins (Pokhrel et al., 2025). Moreover, to reactivate reversibly damaged proteins, HSP90s may act with HOP and HSP100s in stress conditions without HSP70s at all (Blatch and Edkins, 2025) or in complex with them but then with additional co-chaperones.

### HSP110

Among the four members of HSP110 family, HSP105 (HSP110) shows the strongest interaction with HSP70s (Teshima et al., 2021). HSP110 activity has been shown to suppress the synthesis of macroautophagy-related (i.e., lysosomal) factors. It is achieved by downregulating their key transcription factor (named EB). This mechanism is particularly observed in younger individuals (Feleciano et al., 2019). However, this suppression of macroautophagy and the UPS may not be entirely beneficial. For example, a recent study demonstrated that HSP110 overexpression exacerbates amyloid–β (Aβ) aggregate accumulation in *C. elegans* (Montresor et al., 2025). Besides, in a cell culture model for tau aggregation, fragmentation of tau fibrils by the HSP70/40/110 complex resulted in the formation of new tau aggregates (Nachman et al., 2020). Similarly, HSP110-mediated disassembly of prion protein aggregates can lead to the spread of infectious prions (Shoup and Priola, 2025).

### BAG3

Proteins belonging to the B-cell lymphoma-2–associated athanogene (BAG) family also play a role in directing HSP70-mediated proteostasis (Table 2). Among them, BAG3 typically functions as a factor that diverts substrates away from the UPS, often promoting degradation via autophagy. This is primarily relevant for HSC70 (Roperto, 2022), although BAG3 has also been shown to associate with HSPA1A, increasing its activity (Colvin et al., 2014). Although BAG3 is conventionally regarded as proautophagic—and therefore nominally “beneficial” — it may also disrupt proteostasis under certain conditions. For example, BAG3 binding to proteins has been shown to inhibit their proteasomal degradation, and this is either independently of HSP70 levels (Xia et al., 2025) or together with HSP70, as shown by Ruggiero and colleagues reporting the first known inhibitor targeting both BAG3–HSP70 (Ruggiero et al., 2025).

**TABLE 2:** Co-chaperones of HSP70 family. The degree of expression of the effects of various cellular pathways is mediated by the structure, functions of individual co-chaperones and their interaction with HSP70.

Co-chaperone	Human gene nomenclature coding (HGNC) ID	Key features and functions	References
Cytoplasmic co-chaperones			
STIP1 (HOP)	HGNC:11387	Contains three TPR domains (TPR1 and TPR2B binds to HSP70, and TPR2A), two DP domains (DP1 and DP2), and a linker region Preventing protein degradation, folding mediated HSP90 and HSP70	Alvira et al. (2014), Scheufler et al. (2000), Schmid et al. (2012), Brinker et al. (2002), Donnelly et al. (2013)
STUB1 (CHIP)	HGNC:11427	Homodimer, each monomer is composed of an N-terminal TPR domain (binds with the EEVD motif of Hsp70 or Hsp90), a central coiled domain (dimerization of CHIP), and C-terminal U box domain (domain of ubiquitin ligase) Proteasomal degradation	Stankiewicz et al. (2010), Kundrat and Regan (2010)
ST13 (HIP)	HGNC:11343	Dimer, composed of an N-terminal module, tetratricopeptide repeat (TPR) domain, a charged region, GGMP peptide repeats, and a C-terminal domain Stabilizes HSP70, inhibits BAG1	Li Z. et al. (2013), Lüders et al. (2000), Höhfeld and Jentsch (1997)
J-domain proteins, JDPs (HSP40s)			
DNAJA1, A2	HGNC:5229, HGNC:14884	Conserved J domain contains four α-helices, and the second and third helices are connected by linker region Assisting HSP70s (typically, HSC70) in their foldase activity Increasing ATP hydrolysis, thereby accelerating transient association of Hsp70 with client substrates and preventing aggregation Delivering client protein to the substrate-binding site within the β-SBD domain of Hsp70 Ensuring conformation of Hsp70	Mayer and Bukau (2005), Slepenkov and Witt (2002), Venediktov et al. (2023), Kityk et al. (2018)
DNAJB12, 14	HGNC:14891, HGNC:25881		
DNAJC1, 2 (Auxilin 1, 2) DNAJC29 (Sacsin, SACS)	HGNC:20090, HGNC:13192 HGNC:10519		
Nucleotide exchange factors, NEFs			
HSPH1 (HSP105, HSP110)	HGNC:16969	Composed N-terminal domain, S-terminal domain, a linker, and a C-terminal domain Disaggregation client protein, promotes release ADP, inhibits aggregation client protein	Cabrera et al. (2019); Polier et al. (2008), Dragovic et al. (2006)
BAGs	BAG1: HGNC:937 BAG2: HGNC:938 BAG3: HGNC:939 BAG4: HGNC:940 BAG5: HGNC:941 BAG6: HGNC:13919	6 species composed 1 constant domain and 2 variable ones Promoting proteasomal degradation, hydrolysis ATP with HSP40	Kabbage and Dickman (2008), Hantouche et al. (2017), Sondermann et al. (2001)
HYOU1/(GRP170)	HGNC:56704	Composed ATPase domain, β-strand domain, and long loop followed by a helical domain Refolding of denatured protein in the ER and to protect these proteins from proteolysis, binds ATP and imports proteins into the ER	Easton et al. (2000), Saris et al. (1997), Spee et al. (1999)
HSPBP1 (Sil1)	HGNC:24989	Modulates HSP70 activity Inhibits the co-chaperone CHIP Participates in the formation of stress granules	Gowda et al. (2018) Alberti et al. (2004), Mahboubi et al. (2020)

### GRP170

GRP170, a protein that shares structural similarity with GRP78, is well known as a vital ER component. It functions as a nucleotide exchange factor (NEF) for GRP78 and facilitates ER-associated protein degradation, particularly in response to immune stimulation, as detailed in a comprehensive review by Wang and colleagues (Wang et al., 2015). GRP170 is also indispensable for regulating electrolyte metabolism within the ER, as recently demonstrated in podocytes via a GRP170 KO mouse model (Porter et al., 2025).

## HSP70s in autophagy

In contrast to the UPS, autophagy, a lysosome-recruiting type of PQC, involves a transition of damaged proteins through biological membranes via several mechanisms. Some of its molecular actors are common for autophagy as a whole, such as the aforementioned master regulator of autophagy, p62, and a key factor of lysosomal membrane transformation for autophagy, LC3 (Tanida et al., 2004). Many other molecules that participate in autophagy routing are selective receptors (SAR) for cargos to be degraded by lysosomal enzymes (Conway et al., 2020). For HSP70s, chaperone-mediated autophagy via SARs such as lysosome-associated membrane protein 2A (LAMP2A) is rather typical (Venediktov et al., 2023), although HSP70s may also engage additional machinery. Recently, some additional insights into HSP70-assisted autophagy have been reported.

### Aggrephagy

Aggrephagy involves a routing of protein aggregates, which are unable to be degraded by proteasomes owing to their size or generation rate, to lysosomes via specific SARs (Cóppola-Segovia and Reggiori, 2024). In past years, several SARs related to aggrephagy were revealed, and TAX1BP1 was one of the most important, especially in neurons (Sarraf et al., 2020). Known for its multiple functions in and out of cells (Ulrich et al., 2007), TAX1BP1 has recently been shown to participate in aggrephagy only after HSP70 recruitment. Briefly, protein aggregates should bind to the HSP70-HSP40 complex and p97, a powerful ATPase, at the same time and then be recognized by TAX1BP1 and routed into aggrephagosomes (Körner et al., 2025).

### Chaperone-associated selective autophagy

Chaperone-assisted selective autophagy (CASA) mediates lysosomal degradation of proteins delivered by a complex comprising HSC70, BAG3, and HSPB8. The participation of the E3 ubiquitin ligase CHIP is also typically required (Ulbricht et al., 2013). This mechanism primarily facilitates the rapid clearance of large quantities of cytoskeletal proteins—particularly myofibrillar and neurofilament components—and becomes increasingly active compared with the UPS during normal aging (Tedesco et al., 2023). CASA depletion reduces adaptive capacity and accelerates age-related pathology. However, recent data indicate that BAG3-independent autophagic pathways cannot fully compensate for CASA deficiency and may even worsen the proteostatic imbalance. In mice, excessive degradation of soluble proteins such as the gap junction protein connexin 43 was observed under these conditions (Maroli et al., 2024).

### Chaperone-mediated autophagy

Chaperone-mediated autophagy (CMA) requires the lysosomal receptor LAMP2A to recognize and bind KFERQ-like amino acid motifs in client proteins (Kacal et al., 2021; Filali-Mouncef et al., 2022). CMA-accessible proteins account for up to 30% of the cytosolic proteome on the basis of immunodetection techniques and up to 75% according to proteome-wide analyses (Kirchner et al., 2019), including key regulatory molecules such as p53 and glutathione peroxidase 4 (GPX4). CMA is conventionally regarded as a pro-survival mechanism, activated under conditions such as starvation, and dependent on both HSP70s—which bind client proteins—and HSP90s, which stabilize LAMP2A (Tekirdag and Cuervo, 2018). The involvement of HSP70 has recently been confirmed through photo crosslinking experiments showing a direct interaction between HSC70 and KFERQ-like motifs, enabling CMA (Seike et al., 2024). LAMP2A may also bind to other regulatory proteins, such as PARK7, potentially modulating CMA (Zhuang et al., 2025).

Notably, at least in malignant cells, HSP70 has been reported to utilize CMA to promote cell death. A recent study demonstrated that, under heat stress, HSC70 robustly directs GPX4 to CMA, thereby promoting ferroptosis through a marked reduction in GPX4 levels in liver cells (Wang T. et al., 2025). In lung cancer cells, an HSC70/CMA-driven decrease in GPX4 levels is also observed; however, this decrease is accompanied by the downregulation of proferroptotic factors and the upregulation of HSF1 (Peng et al., 2023). On the basis of these findings, Peng and colleagues proposed that HSC70 may act to arrest ferroptosis under certain conditions. However, a reduced GPX4 content was consistently found in both studies. In addition, HSF1 activity is not directly responsible for HSC70 but rather for HSPA1 activity, while the degradation of GPX4 is HSC70 dependent. Thus, there is perhaps no contradiction, and CMA is proferroptotic. This conclusion is reasonable only for HSC70/CMA and not for all HSP70s, as the ER-related machinery with GRP78 is anti-ferroptotic (Yan et al., 2025). Moreover, even HSC70 competes with other chaperones for KFERQ-like motifs. For example, Deng and colleagues reported that the WW domain binding protein 2 can recruit GPX4 earlier than HSC70 and therefore prevent cells from undergoing CMA-driven ferroptosis (Deng et al., 2023).

## Beyond proteostasis

### Exocytosis

HSP70s use a machinery similar to CMA in the formation of exosomes. For this, cooperative recognition of KFERQ-like motifs by LAMP2A and HSP70 is needed, as shown recently for the clearance of the tau protein (Xu et al., 2025). In addition, the binding of HSC70 to clathrin-operating enzyme (cyclin G-associated kinase) has been demonstrated to be the key milestone in the regulation of clathrin-mediated endocytosis (He et al., 2025). Therefore, the cell needs HSP70s both for binding molecules into exosomes and for their insertion into the plasma membrane.

### Extracellular signaling

Even though mostly executing their functions intracellularly, there is a substantial portion of extracellular HSP70s released in endolysosomes, together with such proteins as cathepsin D and LAMP1 with the help of ATP-binding cassette transporters (Mambula et al., 2007), exosomes (Takeuchi et al., 2015) and microvesicles (Komarova et al., 2021). All of these proteins perform various functions, such as stress signaling, immune modulation, and cell-to-cell communication (De Maio and Vazquez, 2013; Kuzmin et al., 2024). For example, vesicle-associated HSP70 has been shown to assist in antigen presentation to CD4^+^ T cells during the immune response (McLaughlin et al., 2010).

### Immune functions

As among the most abundant intracellular proteins, HSP70s serve as signals of compromised cell integrity, namely, damage-associated molecular patterns (DAMPs), when released extracellularly (Kaneko et al., 1997). The DAMP function of HSP70 is so well recognized that the so-called Heck index—the ratio of extracellular to intracellular HSP70 levels—has been widely implemented as a marker of inflammatory status (Soldatov et al., 2024; Kim et al., 2015).

Immune, primarily antigen-presenting cells of myeloid origin as well as nonimmune cells (Qu et al., 2017) perceive extracellular HSP70 signaling via either C-type lectin or scavenger pattern recognition receptors (PRRs), thereby initiating cytokine release involved in innate immune responses (Murshid et al., 2018). Nonetheless, extracellular HSP70 has also been shown to transmit immunosuppressive signals via sialic acid-binding immunoglobulin-like lectin receptors (Siglecs) (Calderwood et al., 2016). Recent studies have shown that both mechanisms can be combined. In cultured cells, Siglec-E receptors form a complex with lectin-like oxidized low-density lipoprotein receptor-1, which simultaneously contains C-lectin and scavenger PRRs (Borges et al., 2025). This signaling operates in an anti-inflammatory mode, reducing the potential of PRRs. Perhaps, the anti-inflammatory modality is also relevant for a lack of damage caused by genetically encoded overload of extracellular HSPA1A in our own studies (Piavchenko et al., 2023).

### Non-immune cell interaction

Moreover, extracellularly released HSP70 exerts signaling activities beyond the immune system. For example, by participating in extracellular signal-regulated kinase-dependent pathways, HSP70 promotes angiogenic activity in cultured endothelial cells (Kim et al., 2016). Inhibition of the release of HSP70-containing exosomes has been recently shown to suppress this pro-angiogenic effect (Wei et al., 2025). Besides, HSPA1, previously known anti-proliferative factor with JAK/STAT pathway involved, is additionally reported to mediate the angiogenic effect of interleukin-28A *in vitro* and *in vivo* via the eNOS/AKT signaling pathway and the activator protein-1/nuclear factor-κB/matrix metalloproteinase-2 (AP-1/NF-κB/MMP-2) cascade (Song et al., 2025). Inhibition of HSP70 in endothelial cells *in vitro* suppresses their proliferation, migration, and vessel formation (Coimbra et al., 2025). Similarly, HSP70 downregulation results in reduced expression of some adhesive proteins (CD31) and cadherins (CD144) which are crucial for both barrier function of the endothelium and intracellular junctions (Pinto-Martinez et al., 2026).

Various HSP70 isoforms are involved in regulating the metabolic activity of adipose tissue. Thus, induction of HSP70 by heat stress enhances lipid accumulation in subcutaneous preadipocytes (Zhang et al., 2024). GRP75 is known as a marker of thermogenic adipocytes (Boucher et al., 2020), and another work has also identified extracellularly released GRP75 as a critical mediator of adipocyte browning (Chen et al., 2024). In contrast, HSC70 in complex with LAMP2A promotes the whitening of brown adipose tissue by mediating the elimination of the thermogenic protein peroxisome proliferator-activated receptor gamma coactivator 1-alpha (PGC1α) via CMA (Zhuang et al., 2025).

### Polynucleotides

HSP70s are responsive to DNA damage (Bailly and Xirodimas, 2021) and participate in control over the proteome by protecting RNA molecules and affecting their kinetics, especially for mRNA (Kishor et al., 2017). HSP70 interact with adenylate-uridylate-rich elements within mRNAs, thereby stabilizing their structure (Kishor et al., 2013). Moreover, HSP70 prevent its own mRNA from proteotoxic stress in hippocampal and spinal neurons by translocation of the mRNA into neurites (Alecki et al., 2024). HSP70 also affect the regulation of noncoding RNA. For example, HSPA1A can upregulate human polymerase III, thereby increasing tRNA synthesis, whereas HSC70, ER-related GRP78 and mitochondrial mortalin do not exert this type of activity (Leone et al., 2024). The transfer of tRNA is also associated with HSP70: in *Saccharomyces cerevisiae*, the molecular chaperones ensure the transportation of tRNA from the cytoplasm to the nucleus (Takano et al., 2015).

In addition, numerous findings confirm that HSP70s control mRNA degradation in an mRNA-specific manner (Walters and Parker, 2015). Moreover, a striking finding of 2025 by Fletcher et al. has shown the direct double-stranded RNA-specific binding capacity of *Drosophila* HSC70-4 (Fletcher et al., 2025), thus implying an antiviral role of HSP70s in the clearance of alien nucleic acids.

## Forward-looking perspectives

Since its discovery, the heat shock protein superfamily has attracted immense interest, fueling many studies revealing its ubiquitous role and powerful therapeutic potential. However, despite the initial high hopes and decades of compelling findings, interest in these molecular chaperones has markedly declined over the past decade. Notably, a similar decline was not observed in the interest of factors such as NF-κB or c-Fos, which were discovered around the same time.

One of the main challenges in developing therapeutic applications targeting HSP70s is their universal involvement in the majority of cellular processes. It is difficult to identify a pathway that is not intertwined with HSP70s. To some extent, HSP70s function as nodes where numerous cellular signaling pathways converge, both in healthy and compromised cells. This may be one of the main reasons that many promising therapeutic strategies targeting HSP70 have failed. Moreover, HSP70 is considered an “undruggable” molecule, reflecting the difficulty of selectively regulating its activity.

However, numerous excellent studies over the past 2 years have revealed outstanding perspectives in almost any cluster of HSP70 employment. For example, the fascinating potential of HSP70s to avoid solidification of altered proteins reveals promising options not only for therapeutic purposes in prevention and treatment of diseases related to protein aggregation but also for engineering of soluble protein compounds such as monoclonal antibodies for diagnosis and theragnostic (Kulkarni et al., 2025). BAG1, facilitating HSP70 activation in the UPS, has been surprisingly shown to change proteasomal conformation, allowing client proteins to enter independent of ubiquinone (Maestro-López et al., 2026).

The intracellular delivery of HSP70 is also a potentially powerful tool for treating neurodegenerative and perhaps cardiovascular diseases. However, such a delivery requires monitoring the intracellular and/or tissue levels and activity of HSPs, the approach that is yet developing (Shapiro et al., 2025; Temezhnikov et al., 2025).

Finally, HSP70 involvement in proteostasis in bacteria, for example, for providing microbial biofilm stability (Matavacas and von Wachenfeldt, 2025), can also be a pharmacological point of action. A similar strategy was recently proposed for the selective inhibition of HSP70 in malaria vector insects, *Anopheles culicifacies* (Goyal et al., 2025). Therefore, a boost-like increase in fundamental knowledge about HSP70 now requires its thorough processing for further implementation in clinical tools.

## Conclusion

Studies of HSP70s seek to transform into an interdisciplinary field of biology, chemistry, and medicine due to the rapidly increasing body of evidence for various HSP70 roles in health and in pathology. Among the most promising directions, one can expect a deeper investigation of HSP70s′ benefits at the boundary between anti-apoptotic (in fact, tumorigenic) potential and proteostasis maintenance in neurodegeneration and aging. We consider future studies should pay attention to immune roles of HSP70s and their effects to control RNA in cells with respect to the findings of the past years.

## Glossary

AKT/PKB: protein kinase B
ALS: amyotrophic lateral sclerosis
AMPK: 5′ adenosine monophosphate-activated protein kinase
AP-1: activator protein-1
ATP: adenosine triphosphate
BACE1: β-site amyloid precursor protein–cleaving enzyme 1
BAG: Bcl-2 associated athanogene family protein
BBB: blood–brain barrier
CASA: chaperone-assisted selective autophagy
CDK1: cyclin-dependent kinase 1
CHIP: C-terminus HSC70-interacting protein
CMA: chaperone-mediated autophagy
CSN: constitutive photomorphogenesis 9 signalosomes
eIF3: eukaryotic initiation factor 3
eNOS: endothelial NO synthase
ER: endoplasmic reticulum
FAF1: FAS-associated factor 1
FIC: cell filamentation proteins
FUS: fused in sarcoma protein
GF: glycine/phenylalanine-rich region of HSP40
GPX4: glutathione peroxidase 4
GRP-E: Gro-P like protein E
GRP78/BiP: glucose-regulated protein 78/binding immunoglobulin protein (HSPA5)
HOP: HSP70/HSP90 organizing protein
HSC70: heat shock cognate protein (HSPA8)
HSF1: heat shock factor 1
HSP40 (eukaryotes)/DNAJ (prokaryotes): 40-kDa heat shock protein family
HSP70/HSPA (eukaryotes)/DNAK (prokaryotes): 70 kDa heat shock protein family
IGFR: insulin-like growth factor receptor
IRE1α: inositol-requiring endoribonuclease 1α
KEAP1: kelch-like enoyl-coenzyme A hydratase-associated protein 1
KO: knockout
LAMP2A: lysosome-associated membrane protein 2A
LC3: microtubule-associated protein 1A/1B-light chain 3
MAPK: mitogen-activated protein kinase
MMP-2: matrix metalloproteinase-2
mTOR: mechanistic/mammalian target of rapamycin kinase
NBD: nucleotide-binding domain, N-terminus
NEF: nucleotide exchange factor
NF-κB: nuclear factor-κB
NRF2: nuclear factor erythroid 2-related factor 2
p97/VCP: valosin-containing protein
PERK: protein kinase RNA-like endoplasmic reticulum kinase
PGC1α: peroxisome proliferator-activated receptor gamma coactivator 1-alpha
PI3K: phosphatidylinositol 3-kinase
PQC: protein quality control
PRRs: pattern recognition receptors
RNA: ribonucleic acid
ROS: reactive oxygen species
SBD: substrate-binding domain
SCF: S-phase kinase-associated protein 1/cullin 1/F-box protein
SERCA: sarcoplasmic reticulum Ca^2+^-ATPase
SIRT1: sirtuin 1, silent information regulator 1
SOD1: superoxide dismutase 1
TARDBP: transactive response DNA binding protein 43 kDa
TAX1BP1: Tax1-binding protein 1
TCAIM: T-cell activation inhibitor in mitochondria
TDP-43: transactive response DNA binding protein of 43 kDa
UPRER: unfolded protein response in the endoplasmic reticulum
UPS: ubiquitin‒proteasome system
YBX1: Y-box binding protein 1
